# Evidence for Acute Myocardial and Skeletal Muscle Injury after Serial Transthoracic Shocks in Healthy Swine

**DOI:** 10.1371/journal.pone.0162245

**Published:** 2016-09-09

**Authors:** Dominik P. Guensch, Janelle Yu, Gobinath Nadeshalingam, Kady Fischer, Jane Shearer, Matthias G. Friedrich

**Affiliations:** 1 Philippa & Marvin Carsley CMR Centre at the Montreal Heart Institute, Montreal, QC, Canada; 2 University Hospital Bern, Department Anaesthesiology and Pain Therapy, Inselspital, University of Bern, Bern, Switzerland; 3 University Hospital Bern, Institute for Diagnostic, Interventional and Paediatric Radiology, Inselspital, University of Bern, Bern, Switzerland; 4 Department of Medicine, McGill University, Montreal, QC, Canada; 5 Department of Radiology, McGill University, Montreal, QC, Canada; 6 Faculty of Kinesiology, University of Calgary, Calgary, AB, Canada; Mount Sinai School of Medicine, UNITED STATES

## Abstract

**Background:**

Previous serological studies have shown controversial results whether defibrillation or cardioversion can cause myocardial injury. Cardiovascular Magnetic Resonance (CMR) can be used to detect myocardial edema, hyperemia and capillary leak as features of acute myocardial injury. The aim of this study was to assess for myocardial and skeletal muscle injury in swine following transthoracic shocks.

**Methods:**

Seventeen anaesthetized swine were examined, with 11 undergoing five synchronized transthoracic shocks (200J). Myocardial and skeletal muscle injury were assessed at baseline and up to 5h post-shock employing T1 mapping, T2 mapping, early and late gadolinium enhancement. Serologic markers (cFABP, TnI, CK, and CK-MB) and myocardial tissue were assessed by standard histology methods.

**Results:**

In myocardial regions within the shock path, T1 and T2 were significantly increased compared to remote myocardium in the same animals. The early gadolinium enhancement ratio between the left-ventricular myocardium and the right pectoral muscle was also increased compared to control animals. After the shocks cFABP and CK were significantly elevated. After shock application, the regions identified as abnormal by CMR showed significantly increased interstitial and myocardial cell areas in histological analysis. This increased cell area suggests significant cellular and interstitial edema.

**Conclusion:**

Our pilot study data indicate that serial defibrillator shocks lead to acute skeletal muscle and myocardial injury. CMR is a useful tool to detect and localize myocardial and skeletal muscle injury early after transthoracic shocks in vivo. In the future the technique could potentially be used as an additional tool for quality control such as verifying insufficient local shock application in non-responders after cardioversion or to develop safer shock forms.

## Background

Electric defibrillation is an important and often life-saving intervention in patients with fatal pulseless and shockable arrhythmia. Electric cardioversion is typically applied for converting atrial fibrillation, flutter and other (supra-)ventricular tachycardia to sinus rhythm.

While its clinical role is well defined, there are no conclusive data on potential injury to the heart or the path of the electrical stimulus through the thorax.

Myocardial injury has been investigated mainly with serologic markers such as troponin (Tn) [1], creatine kinase (CK), myoglobin (Mb), CK-MB [2] and cardiac Fatty Acid Binding Protein (cFABP) [3]. The link between these results and myocardial injury caused by defibrillation or cardioversion, however, has been disputed [4], as some of these markers are not specific to myocardial injury and changes were small. Histologic findings on the other hand provide post-mortem evidence for cardiomyocyte injury [5].

Cardiovascular magnetic resonance imaging (CMR) allows for the in vivo visualization of acute myocardial injury. Acute myocardial injury often features edema, hyperaemia and capillary leak, all which can be detected by CMR using specific sequences [6]. Although CMR is an excellent in vivo assessment of myocardial injury [7], studies on CMR evidence for myocardial injury caused by defibrillation or cardioversion are lacking.

The aim of the present study was to assess for the presence and severity of myocardial injury caused by repeated biphasic synchronized transthoracic shocks.

## Methods

This study was conducted in accordance with the ‘‘Guide to the Care and Use of Experimental Animals” by the Canadian Council on Animal Care. It was approved by the ‘‘Animal Care and Use Board” of the Montreal Heart Institute [#20125003]. Animals were bred at Ferme Lavallée, Quebec, Canada for research and food industry purposes. Animals were brought to the research institute at least 48h before the experiment in groups of 2 or more, and were fed commercial feed (Harlan #7037) twice daily with free access to water up to the start of the experiments.

Seventeen female common landrace swine (32.2±1.3kg body weight) were studied. After premedication (200mg tiletamine, 200mg zolazepam, 0.8mg atropine i.m.) and induction with 2-4mg/kg propofol IV, the animals were intubated. Anaesthesia was maintained with a continuous propofol infusion (12 to 37mg/kg/hr) as required. The femoral artery and vein were cannulated for fluid infusion, blood sampling, as well as invasive blood pressure monitoring. Bolus injections of phenylephrine were administered when required.

The animals were placed in a recumbent position in a clinical 3 Tesla MRI (Magnetom Skyra, Siemens AG, Erlangen, Germany). Using a clinical device (LifePak 15 Defibrillator, Physio-Control, Inc., Redmond, WA, USA), five serial biphasic synchronized transthoracic shocks (200J per shock) were applied to eleven animals within five minutes. Pads were placed on the right pectoral muscle in proximity to the axilla, as well as on the left postero-lateral chest wall anterior of the scapula. Six animals served as a control group. Serology samples and CMR images were obtained at baseline as well as 1h, 3h, and 5h after shock application. At all-time points, cardiac function was measured using standard cine CMR imaging covering the entire left ventricle, and the myocardium was assessed with edema-sensitive native T1 and T2 mapping (Siemens Cardiac Mapping Works in Progress package “Quantitative Cardiac Parameter Mapping T1/T2/T2*”). At the end of the study, 5 hours after shock application, early gadolinium enhancement (EGE) images were obtained prior to and within 3 minutes after i.v. administration of 0.1mmol/kg gadolinium (gadobutrol, Gadovist™, Bayer Healthcare Canada) to assess hyperaemia and capillary leak. Late gadolinium enhancement (LGE) images were obtained 5 and 10 minutes post-contrast respectively to detect scar. Sequence details are in Table 1.

**Table 1:** Imaging Parameters.

Sequence	Function Cine	T1MOLLI	T2Map	EGE	LGE
**Voxel (mm**^**3**^**)**	1.6x1.6x6.0	1.4x1.4x8.0	1.9x1.9x8.0	1.3x1.3.x5.0	1.8x1.8x8.0
**FOV (mm**^**2**^**)**	340x284	360x306	360x288	340x276	340x255
**TE/TR (ms)**	1.43/39.24	1.12/404.96	1.06/238.09	29.0/550	1.09/700
**FA (degrees)**	65°	35°	35°	180°	40°
**BW(Hz/Px)**	962	1085	1184	849	1184
**Imaging Plane**	SAX stack, (8–12 slices)	2 SAX: Basal, Mid	2 SAX: Basal, Mid	2 SAX: Basal, Mid	SAX stack, (8–12 slices)
**Specific Parameters**	ECG-gated SSFP	17 heartbeat (3’5’5), MOdified Look-Locker Inversion recovery, TI minimum = 129s, TI increment = 80 ms	3 SSFP images, adiabatic T2-prep (0, 25, 55ms)	Free-breathing T1-weighted Spin-echo images (averages = 6)	Phase sensitive inversion recovery TI: 280 – 415ms

TE: Echo time, TR: True temporal resolution / repetition time, FA: Flip angle, TI: inversion time, FOV: Field of View: BW: bandwidth, SAX: short-axis, SSFP: steady-state free procession. The T1 mapping sequence uses a modified Look-Locker inversion recovery (MOLLI) [8], optimized in accordance with Kellman et al. 2014 [9] and the T2 mapping is based on the technique proposed by Giri et al. 2009 [10].

Following imaging, animals were euthanized with 200mg propofol and 40mmol KCl IV. A sample of the right pectoral muscle and the entire heart were preserved in 10% formalin. After identifying regions of interest with CMR, myocardial tissue samples were obtained from these segments. All samples were stained according to a standard hematoxylin-eosine protocol.

### Serology

Blood was collected and plasma isolated by centrifugation (3500rpm, 10min, 4°C). Porcine Troponin I (TnI) ELISA-kit (ab174263, Abcam, Cambridge, UK) as well as porcine cardiac fatty acid binding protein (cFABP, ELISA-kit, Cat. No. 2310–5, Life Diagnostics, Inc., West Chester, PA, USA) tests were performed for each sample at baseline and 1, 3 and 5 hours post-shock, according to the operation manuals. Creatine Kinase (CK, clinical standard lab assay) and the porcine cardiac isoform CK-MB (ELISA kit, Cat. No. MBS735380, MyBioSource, San Diego, CA, USA) were measured at baseline and 5h post shock.

### CMR Image Analysis

All images were analyzed using certified evaluation software (cvi^42^, Release 4.1.5, Circle CVI Inc., Calgary, Canada). For cardiac function parameters, epi- and endocardial contours were traced in systole and diastole. For T1 and T2 maps, regions of interest (ROI) were manually defined by tracing visually affected right and left pectoral muscle; epi- and endocardial contours were drawn to include the entire left ventricular myocardium. Additionally, a ROI with a visibly increased T1 or T2 as compared with remote myocardium was selected by the consensus of three readers. These ROI were then compared to remote myocardium in the same animal and to the entire myocardium of the control animals, averaging the T1 or T2 of the ROI or remote tissue respectively of both slices. EGE was calculated by normalizing myocardial signal intensity (SI) to skeletal muscle [6]. For LGE images a visual assessment and an auto-threshold analysis (Otsu method) were performed.

### Histologic Analysis

To quantify cellular edema, the mean area of 5–8 randomly selected cardiomyocytes was calculated with a microscope (Image-Pro plus, MediaCybernetics, version 7.0.0.591 for Windows XP/Vista, Rockville, MD, USA). For assessing changes of the interstitial space fraction of the samples, we performed a semi-quantitative planimetry of three representative areas of each sample slice (ImageJ version 1.46, National Institute of Health, USA).

### Statistical Analysis

Results are displayed as mean values ± SEM. Data was examined for normality with a d’Agostini Pearson omnibus test. For normal distributed data, repeated measures were assessed with a paired t-tests or repeated measures ANOVA with Bonferroni correction if appropriate. Comparisons between the two groups were conducted at each time point using independent t-tests. Otherwise, if the normality test failed, Mann-Whitney U, Friedman’s with Dunn’s post-testing, or Wilcoxon tests were used. Statistical analysis was performed with Graphpad Prism (version 6, Graphpad Inc. San Diego, California, USA). P<0.05 was deemed significant.

## Results

One animal died due to ventricular fibrillation after an un-synchronized shock, resulting in 10 animals with 5 serial shocks and 6 control animals.

### Hemodynamics and Anaesthesia

There was no significant change in heart rate within or between the groups. Six animals in the shock group required phenylephrine support resulting in a mean total dose of 125± 68μg for the group versus control animals in the first 30 minutes after the shocks (p = 0.03) in order to maintain a mean blood pressure >50mmHg. None of the control animals required any vasopressor support.

Propofol requirements of the shocked animals (5430±1148mg) were not different from the control group (6500±1419mg, p = 0.57). Also, there was no difference in fluid regimen with lactated Ringer’s solution between groups (shock 2313±205ml vs. control 2158±216ml p = 0.63).

### CMR Results

#### Functional parameters

There was a small change in end-diastolic volume (Table 2), with borderline significance (p = 0.05) three hours after the shock. Five hours post-shock the steady decrease in end-diastolic volume (p = 0.04) and stroke volume (p = 0.03) from baseline were significant. There was only a statistic trend in the decrease in cardiac output three hours after the shocks (p = 0.06). There were no differences in ejection fraction, end-systolic volume or stroke volume between time-points in the control animals or groups.

**Table 2:** Changes in Cardiac Function Parameters.

		baseline	1h		3h		5h	
**HR**	**Control**	100±6/min	102±3/min	p = 0.20	105±6/min	p = 0.20	107±7/min	p = 0.20
	**Shock**	98±4/min	100±3/min	p = 0.89	98±5/min	p = 0.89	95±6/min	p = 0.89
**ESV**	**Control**	26±1ml	28±2ml	p = 0.12	28±2ml	p = 0.12	30±3ml	p = 0.12
	**Shock**	30±2ml	29±3ml	p = 0.99	30±3ml	p = 0.99	28±3ml	p = 0.99
**EDV**	**Control**	62±4ml	66±4ml	p = 0.57	66+3ml	p = 0.57	66+2 ml	p = 0.57
	**Shock**	72±4ml	70±4ml	p = 0.62	67±4.ml	**p = 0.05**	63 ±4ml	*p = 0.04
**SV**	**Control**	36±4ml	39±4ml	p = 0.86	37±2ml	p = 0.86	36±2ml	p = 0.86
	**Shock**	42±3ml	41±3ml	p = 0.99	37±3ml	**p = 0.21**	35±2ml	*p = 0.03
**EF**	**Control**	58±4%	58±4%	p = 0.37	58±3%	p = 0.37	55±4%	p = 0.37
	**Shock**	58±4%	59±3%	p = 0.79	58±3%	p = 0.79	55±3%	p = 0.79
**CO**	**Control**	3.6±0.4L/min	3.9±0.4L/min	p = 0.96	4.0±0.4L/min	p = 0.96	3.8±0.3 L/min	p = 0.96
	**Shock**	3.9±0.2L/min	4.1±0.3L/min	p = 1.00	3.6±0.2L/min	**p = 0.06**	3.2±0.2L/min	p = 0.22
**Mass (sys)**	**Control**	62±5g	60±2g	p = 0.58	59±2g	p = 0.58	59±3g	p = 0.58
	**Shock**	58±2g	60±3g	p = 0.90	61±4g	p = 0.90	57±4g	p = 0.90

There was a decrease in end-diastolic volume (EDV) in the shocked animals, which was significant at 5 hours after the shocks. There was also a non-significant trend for a decreased cardiac output (CO) observed 3 hours post-shock, but this was not observed after 5 hours. Stroke volume (SV) was decreased 5 hours post-shock. In the control animals there was no change in any of the function parameters assessed with CMR.

#### T1 and T2 mapping

**Fig 1:**
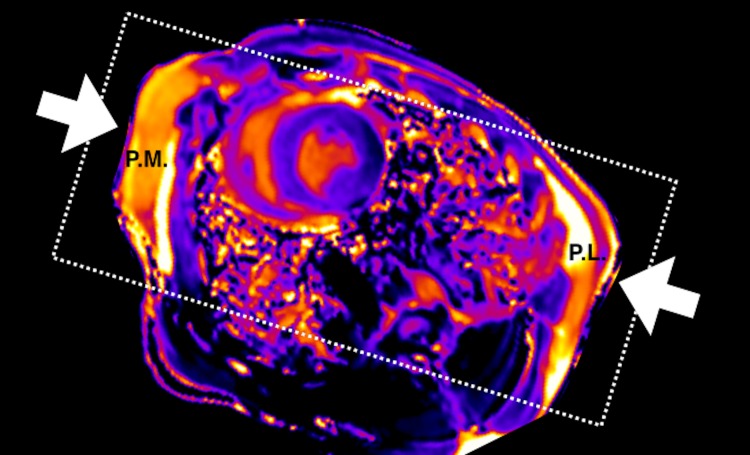
Shock path and pad location (arrows) shown by increased T1 in the right pectoral muscle (p.m.) and the postero-lateral pad location (p.l.) in a mid-ventricular slice.

Defibrillation pad locations were visible by increased T1 (Fig 1) and T2 in the images.

**Fig 2:**
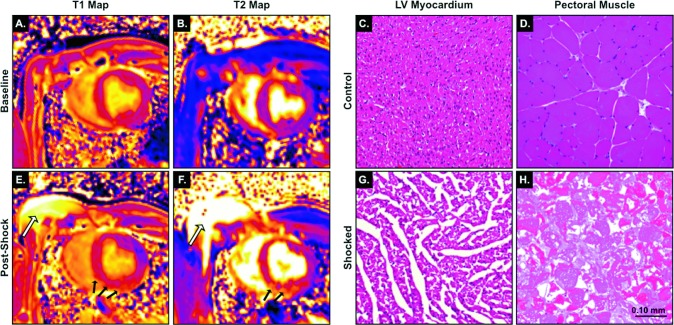
T1 maps and T2 maps of a mid-ventricular slice before **(A, B)** and the shocks **(E, F):** There is evidence for skeletal muscle edema under the pads (white arrow). In the myocardium, an increased T1 and T2 are apparent in the inferior and septal wall of the LV (black arrows). While, the hematoxylin-eosin stains of the LV myocardium and right pectoral muscle of control animals show normal cell and tissue configuration **(C, D)**, the ROI-selected LV samples exhibit signs of interstitial edema in the shocked animal **(G)**. Additionally, the skeletal muscle under the pad **(H)** shows signs of muscle fibre necrosis (scale bar = 0.10mm).

In all shocked pigs, a marked regional increase in native T1 and T2 values was detected, located in the anterior and inferior wall (n = 8), in the septum and inferior wall (Fig 2, n = 1) and in the septum and lateral wall (n = 1) of the left ventricle, corresponding to defibrillation pad locations. These regions were selected as ROI for quantitative analysis.

**Fig 3:**
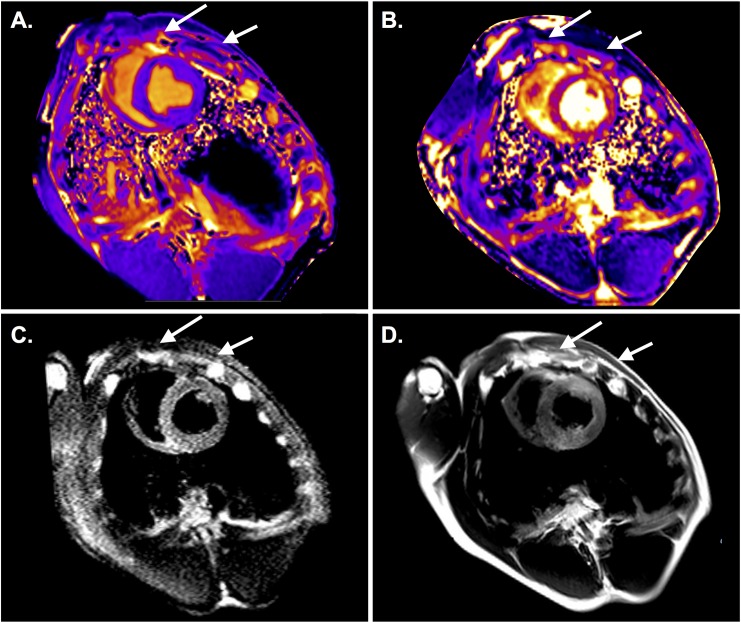
Animal with aberrant shock path over the thoracic wall through the minor pectoral muscle (arrows) and the intercostal muscles indicated by T1 (A) and T2 maps (B), as well as a T2 STIR (C) in the basal slice. This animal did not only show a global EGE increase, but regional abnormalities in the anterior and inferior wall (D).

Interestingly, one animal showed an abnormal path of tissue changes compared to the other animals. Instead, in this animal T1 and T2 visually increased in the minor pectoral muscle and the intercostal muscles over the thorax, indicating that most of the current may have passed over the chest wall. In the anterior wall increased T2 was observed (Fig 3).

#### Quantitative analysis

**Fig 4:**
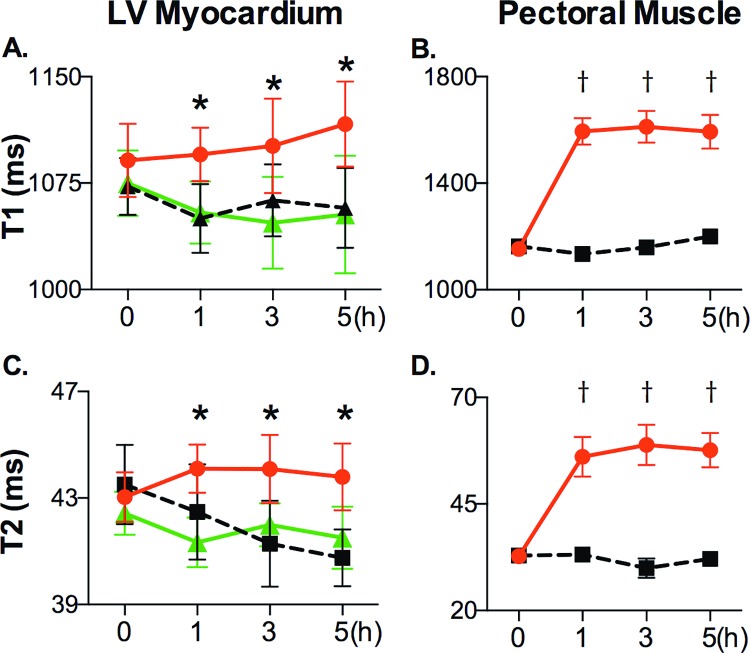
Time course of T1 and T2 after application of transthoracic shocks: In the affected regions (red circle), T1 **(A)** and T2 **(B)** were significantly increased after shock application, compared to remote myocardium in the same animals (green triangle). T1 **(C)** and T2 **(D)** were significantly increased in the right pectoral muscle (red circle) compared to the control animals (black square) and to their own baselines. (*p<0.05 between ROI and remote myocardium, †p<0.01 in pectoral muscle of shocked animals vs. baseline and vs. control animals).

In the quantitative analysis there was no significant difference between native T1 of the ROI of the shocked animals and the controls (p>0.17), but we observed variation in T1 in the control animals (Fig 4A). However, T1 in the affected ROI was significantly higher than that of the remote myocardium in the same animals (p<0.05) after shock application. In the right pectoral muscle, T1 increased after the shocks and remained elevated throughout the study (Fig 4B, *P*<0.01).

T2 of the ROI was significantly longer post shock than in remote myocardium of the same animals (*P*<0.05, Fig 4C). Neither affected regions (p>0.14) nor remote T2 were significantly different from those of control animals (p>0.26). Myocardial T2 post-shock showed a trend to be higher than the individual pre-shock baselines (p = 0.06). As in the T1 images, the right pectoral muscle showed a significantly longer T2 than that of healthy control animals (p<0.01, Fig 4D) post shock.

#### Early Gadolinium Enhancement (EGE)

**Fig 5:**
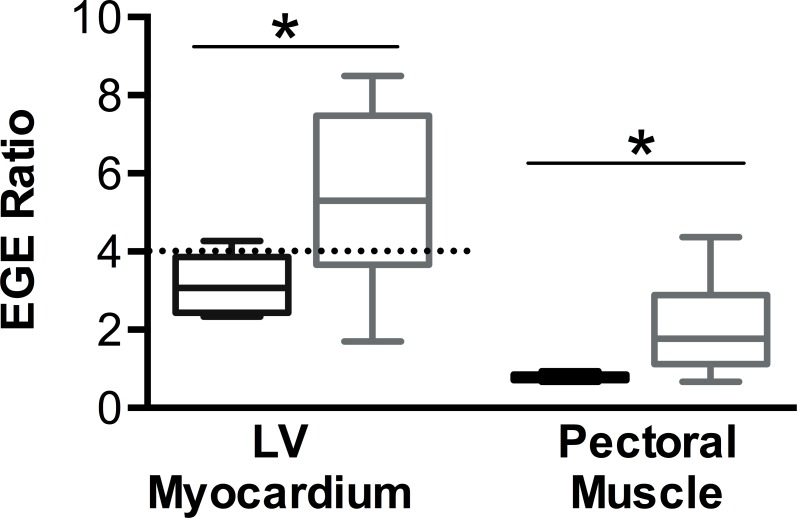
EGE Ratio was significantly higher in the right pectoral muscle and in the LV in the shocked animals (grey) than the control group (black), *p<0.05. The EGE ratio in the LV was above the pathologic threshold >4 as defined in human studies.

The EGE ratio (myocardial enhancement divided by healthy skeletal muscle enhancement) was significantly higher in the global LV myocardium and the right pectoral muscle of the shocked pigs than in the controls (p<0.05, Fig 5). The increased EGE ratio in the LV myocardium was global in most animals, while two animals additionally showed a regional increase.

#### Late Gadolinium Enhancement (LGE)

No animal showed signs for pre-existing scar or fibrosis in LGE images.

### Histology

**Fig 6:**
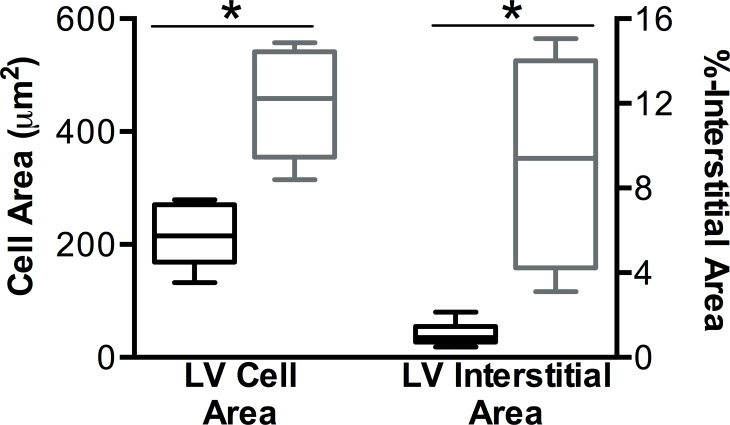
The myocardial cell area in SAX muscle fibers and the interstitial area in the histology samples of affected regions of the shocked animals (grey, n = 7) as defined by an increased T1 or T2 versus control animals (black, n = 6). Both, LV cell area and %interstitial space were found to be greater in the samples of the shocked animals than in the controls *p<0.01.

The mean interstitial space in LV myocardial tissue obtained from regions with abnormal T1 or T2 was larger in pigs of the shock group than in control animals (9.4±1.7% vs. 1.1±0.2%; p*<*0.01; Fig 2 and Fig 6).

Cell area of the cardiomyocytes was increased after the shocks (443±35μm^2^ vs. 219±26μm^2^ in the control animals; p<0.01; Fig 2 and Fig 6).

In the shocked animals, there was destruction of the majority of skeletal muscle cell fibres in the specimens (Fig 2). In the shock group samples of three animals were not available resulting in only seven specimens.

### Serology

**Fig 7:**
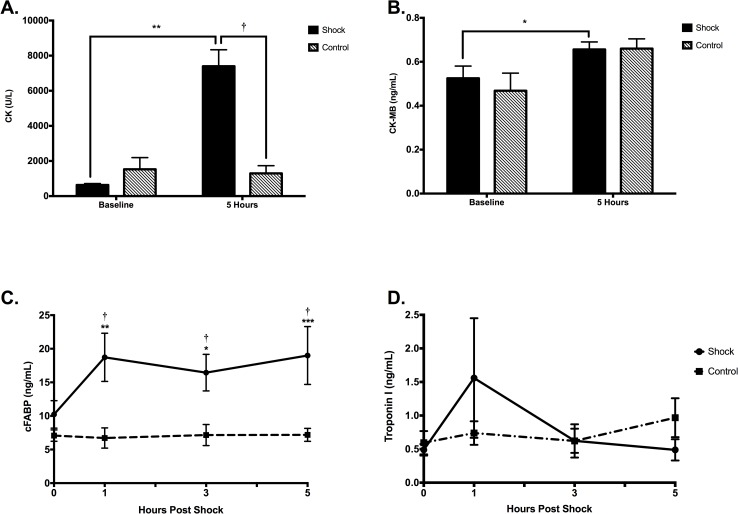
Serology Results. Changes in cFABP, TnI, CK and CK-MB in control animals and the shock group at baseline and 1, 3 and 5h observation time. *P<0.05, ** *p*<0.01, ***p<0.001 versus baseline. †*P*<0.01 at the same time-points between the groups. A significant increase is seen in cFABP and CK compared to baseline in the shocked animals, which were also higher than the corresponding levels of the control group. CK-MB was also found to be significantly higher in the shocked animals compared to baseline, with a trend of being higher after 5 hours of observation compared to its baseline in the control group (p = 0.06). Other parameters compared to baseline or control group p>0.23.

Porcine cFABP was significantly increased at all-time points after the shock compared to baseline (p<0.05), as well as compared to the control animals (p<0.01, Fig 7). Total CK was significantly increased 5h post shock compared to baseline (p = 0.002) and to the control animals at 5h (p = 0.001). CK-MB also showed an increase in the shocked animals after 5h (p = 0.03). There was no difference in porcine TnI.

## Discussion

To our knowledge we have detected and reliably localized acute myocardial and skeletal muscle injury after serial transthoracic shocks in-vivo the first time using a comprehensive CMR study with state-of the-art clinical sequences. The presence and extent of acute injury detected by CMR was verified by histology and serology.

Furthermore, our study indicates a utility of CMR to detect skeletal muscle and myocardial injury after serial transthoracic shocks in vivo, potentially to detect insufficient shock application (non-responders after cardioversion) or to further improve the devices (different shock forms) and protocols (same energies vs. increasing energies; stacked vs. single shocks).

All these sequences are currently used for human assessment, and thus our technique has a high potential to be translated into clinical use.

### CMR

As recent serologic studies failed to indisputably show myocardial injury, we used several CMR techniques to not only detect but also localize the injury caused by serial transthoracic shocks. First, we observed an increased early gadolinium enhancement ratio in the LV myocardium of animals with shock application. This is consistent with previously published data from human studies, where an EGE ratio of 4 or more indicates hyperaemia and capillary leak in inflammatory myocardial disease [11]. Furthermore, the mapping data show an increase in both native T1 and T2 values after transthoracic shocks in the ROI only compared to the remote myocardium, reflecting the longer T1 and T2 of tissue with edema [10,12–15].

Of note, the increase of T1 and T2 in the affected regions did not reach statistical significance when compared to control animals, which may be due to the small sample size. The visible decline in T1 and T2 in the control animals, as well as in the remote myocardium may be a result of too restrictive fluid administration during the experiments in both groups.

The significant injury of the right pectoral muscle from both the T1 and T2 analyses after the shocks show electroporetic skeletal muscle injury [16,17] that is paralleled by the increase in CK activity.

We found a decrease in EDV and a trend towards a lower cardiac output after shock administration. We interpret these findings as reflective of myocardial edema with increased stiffness and resulting impaired left-ventricular filling. The absence of late gadolinium enhancement excludes pre-existing myocardial injury.

### Serology

Cardiac troponins are sensitive serologic markers for myocardial damage. Current literature does not conclusively support that the application of transthoracic electric shocks leads to myocardial injury. While there is data indicating an elevation in serum troponin after transthoracic shocks, most of these studies show only small and clinically insignificant increases [1,18]. In a meta-analysis of studies after external cardioversions only 7 of 442 patients and 1 of 368 patients showed an increase in cardiac Troponin I and T, respectively [19]. Additionally, myoglobin levels and creatine kinase (CK) activity are non-specific markers for muscle injury and an increase in these markers after transthoracic shocks therefore is not reliable proof of myocardial injury and may be also attributed to skeletal muscle injury [2,4,20].

In accordance with existing literature, we did not find changes in porcine TnI levels. In the context of our findings and current literature we doubt that cardiac troponin assays are suitable markers to assess acute myocardial electroporation injury. However, more data is needed to support this assumption. The increase in CK activity in our study may be partially related to myocardial injury but are expected to be mainly due to skeletal muscle injury.

Cardiac Fatty Acid Binding Protein (cFABP) is a marker that is elevated early after myocardial injury and after transthoracic shocks [3,21], and interestingly is a sensitive marker to injury in the absence of necrosis [22]. The persistent elevation of cFABP after the shocks confirms the high sensitivity of this marker to detect myocardial electroporation injury, even late after the shock application.

### Histology

Compared to serologic markers, histology is a more reliable test for the detection and localization of tissue injury, if the sample was taken from truly affected tissue. We show that histologic samples which were regionally matched with abnormal tissue characteristics in CMR images, had an increased proportion of interstitial space in comparison to samples of control animals. This increase may represent capillary leakage, resulting in edema and longer native T1 and T2 times. We also found evidence for intracellular edema shown by an increased cell area in the shocked pigs, likely caused by electroporation injury of the cellular membranes and the subsequent water influx.

Skeletal muscle injury was much more pronounced than the myocardial injury, as seen by higher T1 and T2, suggesting that the skeletal muscle under the defibrillation pad significantly scavenges and attenuates the energy that reaches the myocardium. Similarly, in the histology samples, disruption of cell integrity was observed in the right pectoral muscles of the shocked animals whereas the cardiomyocytes only exhibited signs of cellular edema.

### Limitations

The aim of this pilot study was to test the feasibility of CMR to detect shock related injuries. Considering the weight of the animals (32.2±1.3kg), relatively high cumulative energies were used (>4J/kg per shock). However, guidelines stipulate that when using an automated external defibrillator, adult pads are to be used in children, when paediatric pads are not available [23,24]. The AHA guidelines even allow energies for subsequent shocks up to 10J/kg in children. With 200J per shock we lie within the maximum propagated dose suggested in the paediatric guidelines. Five consecutive shocks during a resuscitation effort are not exceptional and may be realistic in many clinical scenarios. Especially the new concept of stacked shocks when a deterioration into ventricular fibrillation is witnessed during a percutaneous coronary intervention or the concept of escalating energies make our investigated cumulative dose of 1kJ clinically relevant. Our data may not be applicable to single shocks, where there is likely less damage. Future CMR studies need to investigate a dose relationship. In this study we only assessed acute injury such as myocardial edema, and hyperaemia and changes in short term function parameters. Further our data do not include follow-up scans later than 5 hours to assess a chronic injury. In our model we used healthy animals without pre-existing heart disease as a possible confounder and different results may occur in diseased hearts. As the shock pathway differed between the subjects, ROI were visually chosen by the consensus of three readers; yet a selection bias cannot be reliably excluded. It is also important to note that this pilot study had a small sample size. Further studies have to be undertaken to address specific outcomes such as function parameters, edema or hyperaemia.”.

## Conclusion

Our data indicate that serial applications of defibrillator shocks can lead to acute skeletal muscle and myocardial injury detectable by CMR scans in-vivo. In the future the technique could potentially be used as an additional tool for quality control such as verifying insufficient local shock application in non-responders after cardioversion or to develop safer shock forms. There is suspicion by clinicians that a fraction of all applied shocks does not correctly through the desired route through the chest, therefore such a diagnostic tool may indeed be helpful.

## Supporting Information

S1 File Raw data is provided for all reported values in Microsoft Excel format, with one worksheet per data field. (XLSX)
